# Quasi-static compression test dataset of sinusoidally-corrugated multi-cell PLA+ energy absorbers fabricated by fused deposition modeling (FDM)

**DOI:** 10.1016/j.dib.2026.113173

**Published:** 2026-08-14

**Authors:** Ahmed Saber, Murat Altin, Erdem Acar, Mehmet Ali Güler

**Affiliations:** a College of Engineering and Technology, American University of the Middle East, Egaila 54200, Kuwait; b Department of Automotive Engineering, Gazi University, Ankara 06590, Türkiye; c TOBB University of Economics and Technology, Ankara 06560, Türkiye

**Keywords:** Additive manufacturing, Crashworthiness, Latin hypercube sampling (LHS), Machine learning (ML), Surrogate modeling

## Abstract

This article presents an experimental dataset for additively manufactured, sinusoidally-corrugated multi-cell (SCMC) energy absorbers tested under quasi-static axial compression. Forty SCMC configurations were generated by Latin Hypercube Sampling (LHS) over four geometric design variables, together with a nominal configuration (N-SCMC) and a simple tube without corrugation (STWC) as baseline designs. All specimens were fabricated from PLA+ filament on a Bambu Lab A1 Mini fused deposition modeling (FDM) printer, and the PLA+ mechanical behavior was characterized by uniaxial tensile testing in accordance with ASTM D638. The two baseline configurations were tested in triplicate to confirm repeatability. The dataset comprises PLA+ tensile characterization data, geometric design parameters, STL geometry files, axial force–displacement responses, and derived crashworthiness indicators such as the crush force efficiency (CFE), and specific energy absorption (SEA), along with sequential deformation images captured at 5 mm displacement intervals. These data support surrogate modeling, machine learning applications, optimization, and validation of numerical models for FDM-printed polymer energy absorbers.

Specifications Table

**Table:** 

Subject	Engineering & Materials science
Specific subject area	Quasi-static axial crushing of FDM-printed sinusoidally-corrugated multi-cell PLA+ energy absorbers for crashworthiness evaluation.
Type of data	Processed, Raw, Image, Table
Data collection	Design configurations were generated by Latin Hypercube Sampling (LHS) with the MATLAB “lhsdesign” function. Specimens were modeled in SOLIDWORKS, exported as STL files and printed from eSUN 1.75 mm PLA+ filament on a Bambu Lab A1 Mini FDM printer. The PLA+ was characterized by uniaxial tensile testing (ASTM D638) on a Salt ST-1002 machine (50 kN) at 5 mm/min. Quasi-static axial compression tests used a Tinius Olsen 50ST machine (50 kN) at 5 mm/min up to 50 mm displacement. Force–displacement data were exported as .xlsx files, images were captured at 5 mm intervals, and crashworthiness indicators were computed in Microsoft Excel.
Data source location	Institution: American University of the Middle East City: Al Ahmadi Country: Kuwait
Data accessibility	Repository name: Mendeley Data Data identification number: 10.17632/v7p6y654ft.4 Direct URL to data: https://data.mendeley.com/datasets/v7p6y654ft/4
Related research article	M.A. Güler, A. Saber, M. Altin, E. Acar, Design and optimization of additively manufactured sinusoidally-corrugated multi-cell energy absorbers, Mater. Test. (2026). https://doi.org/10.1515/mt-2026–0038

## 1 Value of the Data

• These data are valuable to researchers in additive manufacturing (AM) and structural crashworthiness. The dataset provides experimentally measured force–displacement data, derived crashworthiness indicators, sequential deformation images, and Standard Tessellation Language (STL) geometry files for 40 sinusoidally-corrugated multi-cell (SCMC) energy absorbers fabricated by fused deposition modeling (FDM) and designed using Latin Hypercube Sampling (LHS), along with a nominal SCMC configuration (N-SCMC) and a simple tube without corrugation (STWC). Open experimental datasets of this kind remain scarce for polymer-based corrugated multi-cell structures.
• These data can be used to train, validate, and compare different surrogate models, including polynomial response surfaces (PRS), radial basis functions (RBF), and Kriging (KRG), as well as machine learning approaches such as artificial neural networks (ANN), Gaussian process regression (GPR), and support vector regression (SVR), for the prediction of crashworthiness indicators and design optimization of FDM-printed energy absorbers, using the provided geometric design variables and experimentally measured responses.
• The force–displacement data, derived crashworthiness indicators, and sequential deformation images can serve as a benchmark for researchers developing or validating numerical models of additively manufactured polymer energy absorbers, enabling direct quantitative and qualitative comparison against numerical model predictions.
• The STL geometry files enable direct fabrication of the tested specimens, allowing independent experimental replication and further investigation of the reported crashworthiness data without the need for CAD reconstruction.

## 2 Background

Vehicle safety depends on structural components' ability to manage impact energy during collisions, a property known as crashworthiness. Energy absorbers achieve this through progressive deformation under axial loading, converting kinetic energy into strain energy and reducing transmitted force. Crashworthiness performance depends on material selection and geometric design. AM enables the fabrication of complex geometries unattainable through conventional methods, enhancing energy absorption efficiency. FDM is widely adopted for polymer-based energy absorbers, valued for its material compatibility, ease of use, and low cost [1]. Among available polymers, PLA+ has shown strong potential for energy absorption due to its processability and crashworthiness [2,3]. Multi-cell tubular geometries provide superior energy absorption over single-cell designs [4] but are prone to unstable collapse and high peak forces [5]. Sinusoidally-corrugated profiles address these limitations through stable, progressive folding with reduced peak loading [6]. Combining sinusoidal corrugation with multi-cell architecture yields SCMC energy absorbers that leverage both strategies. In the associated research article [7], the N-SCMC configuration improved specific energy absorption (SEA) and crush force efficiency (CFE) by 13% and 77%, respectively, over STWC configuration, and a machine-learning-based optimization raised these improvements to 55% and 82% over the STWC configuration. This data article presents the corresponding experimental dataset, including PLA+ material characterization data, geometric design parameters, STL files, force–displacement data, crashworthiness indicators, and deformation images. Beyond supporting the associated research article, this dataset provides a standalone resource for surrogate modeling, machine learning, optimization, and numerical model validation for FDM-printed energy absorbers.

## 3 Data Description

**Fig. 1:**
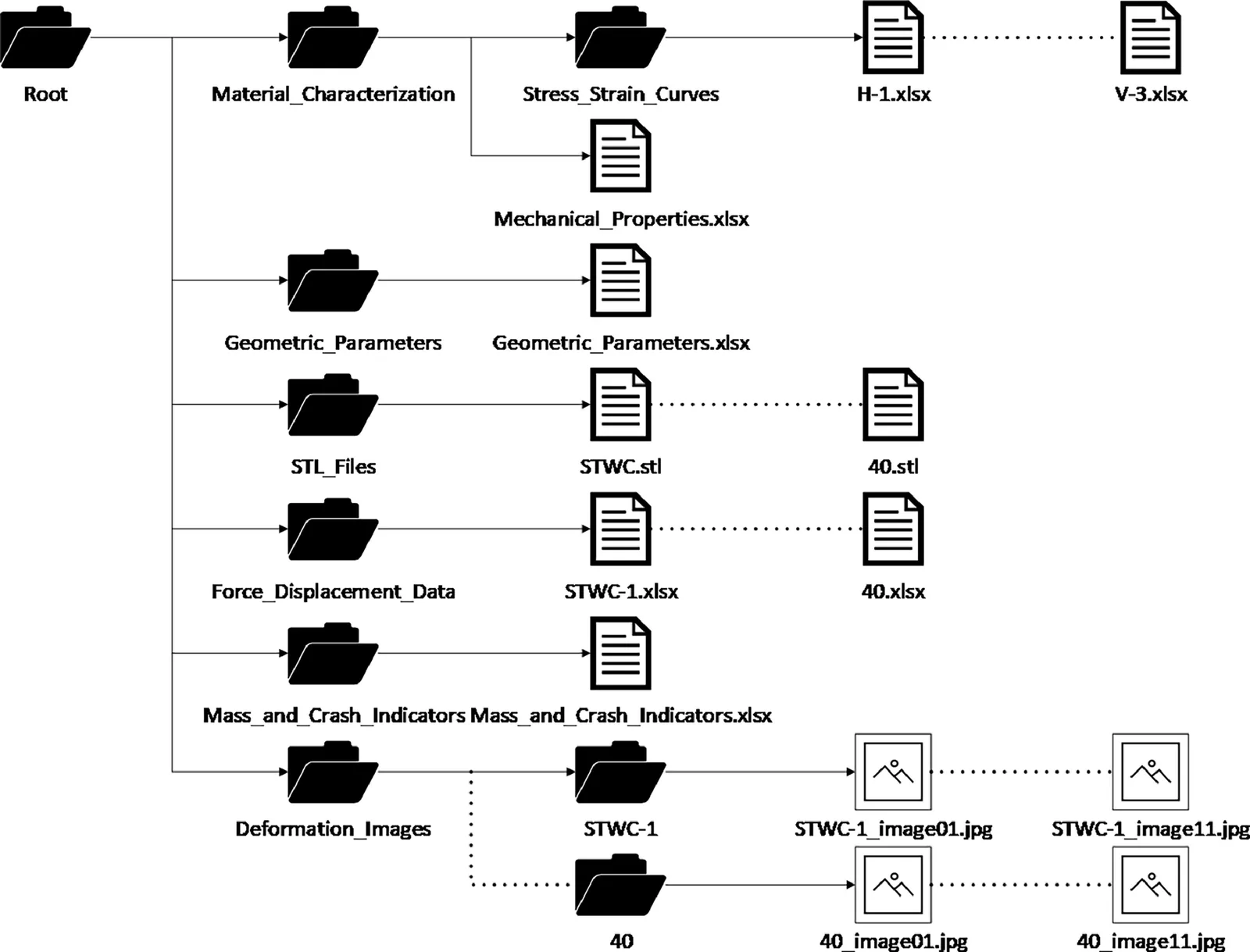
Dataset organization.

The dataset is organized into six top-level folders, as illustrated in Fig. 1.

The Material_Characterization folder contains PLA+ material characterization data. The Stress_Strain_Curves subfolder includes six Excel files: three for specimens printed in the horizontal (H) orientation (H-1.xlsx, H-2.xlsx, H-3.xlsx) and three for specimens printed in the vertical (V) orientation (V-1.xlsx, V-2.xlsx, V-3.xlsx). Each file contains two columns: engineering strain (mm/mm) and engineering stress (MPa). The Mechanical_Properties.xlsx file summarizes the derived mechanical properties for all six tensile specimens, including density (g/cm³), Young's modulus (MPa), yield strength (MPa), and ultimate tensile strength (MPa). The mean and standard deviation of each property are also reported, together with the p-value of a two-sample *t*-test assuming unequal variances, comparing the horizontal and vertical orientations.

The Geometric_Parameters folder contains a single file, Geometric_Parameters.xlsx, which lists the four geometric design variables for the nominal N-SCMC configuration and all 40 LHS-generated SCMC configurations: the inner tube outer diameter (D) (mm), web count of the multi-cell core (W), longitudinal half-wave count (N), and corrugation amplitude (A) (mm).

The STL_Files folder contains 42 STL geometry files representing the surface geometry of each unique configuration: STWC.stl and N-SCMC.stl for the two reference configurations, and 01.stl through 40.stl for the 40 LHS-generated SCMC configurations.

The Force_Displacement_Data folder contains 46 Excel files providing axial force versus displacement data recorded during quasi-static compression testing. The STWC and N-SCMC configurations were each tested in triplicate, corresponding to files STWC-1.xlsx through STWC-3.xlsx and N-SCMC-1.xlsx through N-SCMC-3.xlsx, respectively. The 40 LHS-generated SCMC configurations are provided as 01.xlsx through 40.xlsx. Each file contains two columns: crosshead displacement (mm) and axial compressive force (kN).

The Mass_and_Crash_Indicators folder contains a single file, Mass_and_Crash_Indicators.xlsx, which reports the specimen mass and derived crashworthiness indicators for all 46 tested specimens. The reported quantities are: specimen mass (m) (g), effective crush displacement $\left(\delta_{max}\right)$ (mm), crushing efficiency (CE), total energy absorbed $\left(E_{T}\right)$ (kJ), peak crushing force (PCF) (kN), mean crushing force (MCF) (kN), SEA (kJ/kg), and CFE. The mean and standard deviation of each quantity are also reported for the two baseline configurations (STWC and N-SCMC), each tested in triplicate.

The Deformation_Images folder contains 46 subfolders, one per tested specimen. The STWC and N-SCMC configurations are each represented by three subfolders (STWC-1, STWC-2, STWC-3 and N-SCMC-1, N-SCMC-2, N-SCMC-3, respectively), and the 40 LHS-generated configurations are represented by subfolders 01 through 40. Each subfolder contains 11 sequential images captured at 5 mm displacement intervals throughout the compression test, provided in JPG format and named sequentially from image01 to image11, prefixed by the specimen’s name (e.g., STWC-1_image01.jpg through STWC-1_image11.jpg).

## 4 Experimental Design, Materials and Methods

### 4.1 Experimental design

**Fig. 2:**
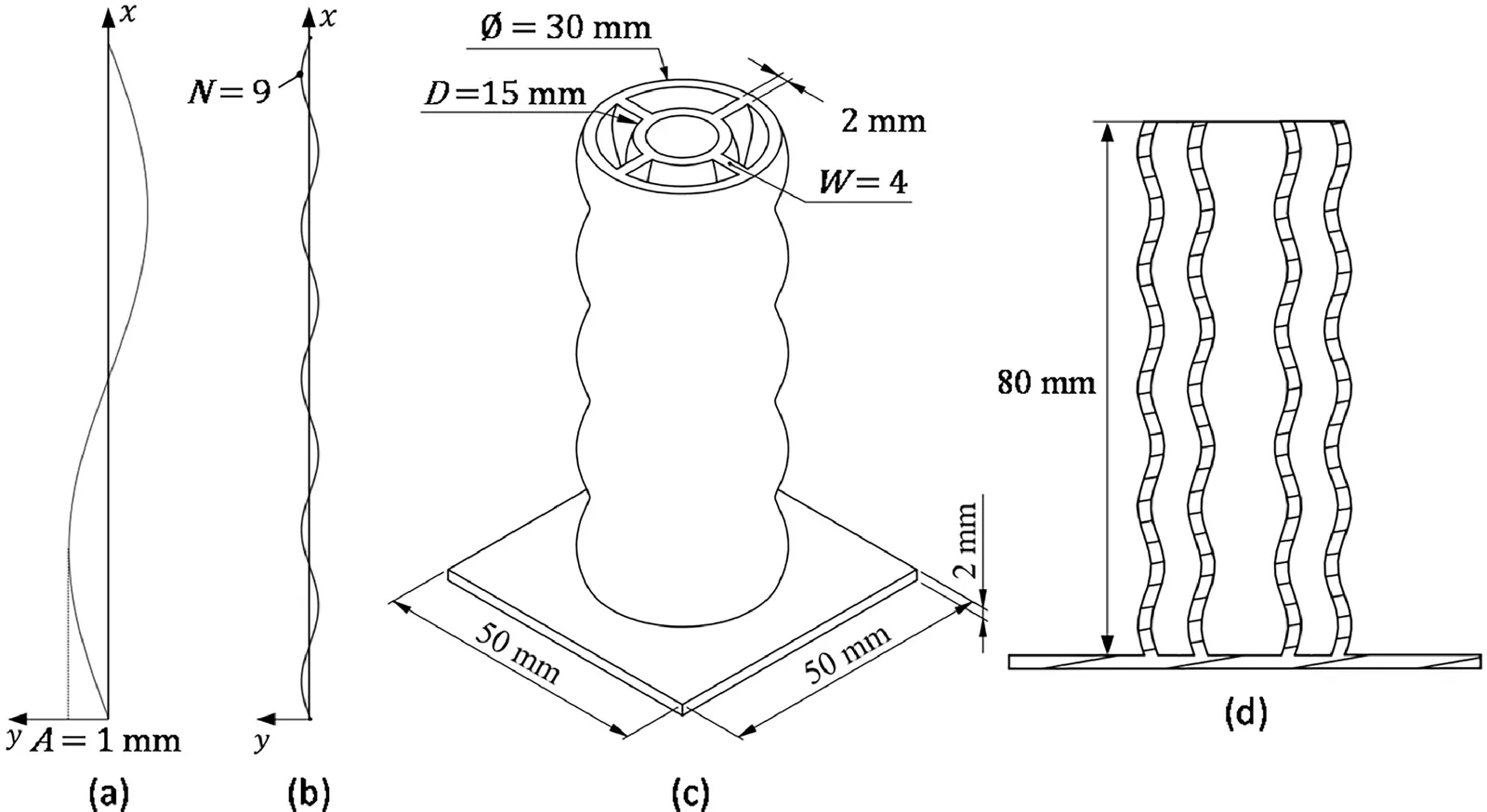
Geometric features of SCMC: (a) sinusoidal wave profile at A = 1 mm; (b) sinusoidal wave profile at N = 9; (c) N-SCMC isometric view; (d) N-SCMC section view.

The sinusoidal corrugation imposed on the energy absorber wall follows the wave profile shown in Fig. 2, and is expressed as:

(1) $$y=A\;\text{sin}\left(\frac{N\pi x}{L}\right)$$

where A is the corrugation amplitude, N is the longitudinal half-wave count, and L is the length. For N-SCMC, N = 9, and A = 1 mm, as shown in Fig. 2a and Fig. 2b Both the inner and outer walls carry this same profile, so they fold together during collapse. The remaining geometric variables are D and W, as shown in Fig. 2c. For the nominal configuration, D = 15 mm and W = 4. The outer tube diameter, wall thickness, and total length were held fixed at 30 mm, 2 mm, and 80 mm, respectively, in accordance with an earlier work in the literature [8]. To suppress slipping under axial load, a 50 mm × 50 mm × 2 mm base plate was attached beneath each specimen [1], as illustrated in Fig. 2c. The internal arrangement of the N-SCMC structure is presented in Fig. 2d

The SCMC geometry is governed by four design variables. The first, D, ranges from 10 to 20 mm; the second, W, from 2 to 6; the third, N, from 7 to 11; and the fourth, A, from 0.5 to 1.5 mm. Within these ranges, LHS was applied through the MATLAB “lhsdesign” function to generate 40 configurations, equal to ten samples per design variable. A sampling density of this order is widely regarded as sufficient for constructing surrogate and machine-learning models of appropriate precision while keeping the experimental effort doable [5]. In this method, the range of each variable is partitioned into equal-probability intervals from which a single value is drawn, producing uniform, space-filling coverage of the design domain. A thorough description of the technique is given by Park [9].

## 5 Materials

**Fig. 3:**
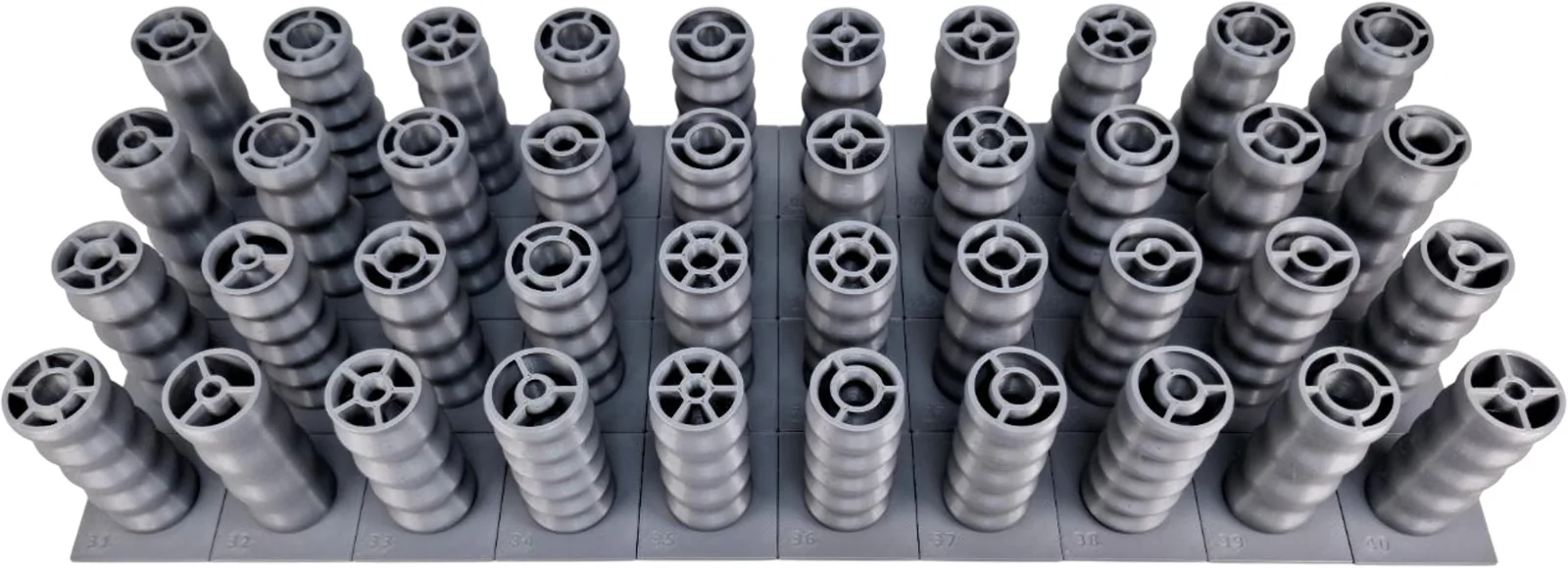
40 manufactured SCMC energy absorbers.

All specimens were manufactured from eSUN 1.75 mm PLA+ filament. Each geometry was modeled in SOLIDWORKS with no dimensional tolerances specified, exported in STL format, and sliced in Bambu Studio to obtain the G-code, with no shrinkage or scaling compensation applied. Printing was conducted on a Bambu Lab A1 Mini FDM printer using the parameters listed in Table 1, which follow the filament and printer manufacturers’ recommendations for reliable mechanical performance and print quality. Specimens were printed in the vertical orientation, which aligns the build direction with the axial loading direction, consistent with previous FDM tubular energy absorber studies [10,11]. The seam position was set to random so that no weak line, formed where successive layers start and end, persisted along the tube wall as a stress concentrator. Fig. 3 shows the 40 manufactured SCMC energy absorbers.

**Table 1:** Print parainmeters.

Print Parameters	Value

Infill density (%)	100
Diameter of nozzle (mm)	0.6
Layer height (mm)	0.3
Bed temperature ( °C)	65
Printing temperature ( °C)	220
Inner Wall Print speed (mm/s)	150
Outer Wall Print speed (mm/s)	120

## 6 Methods

**Fig. 4:**
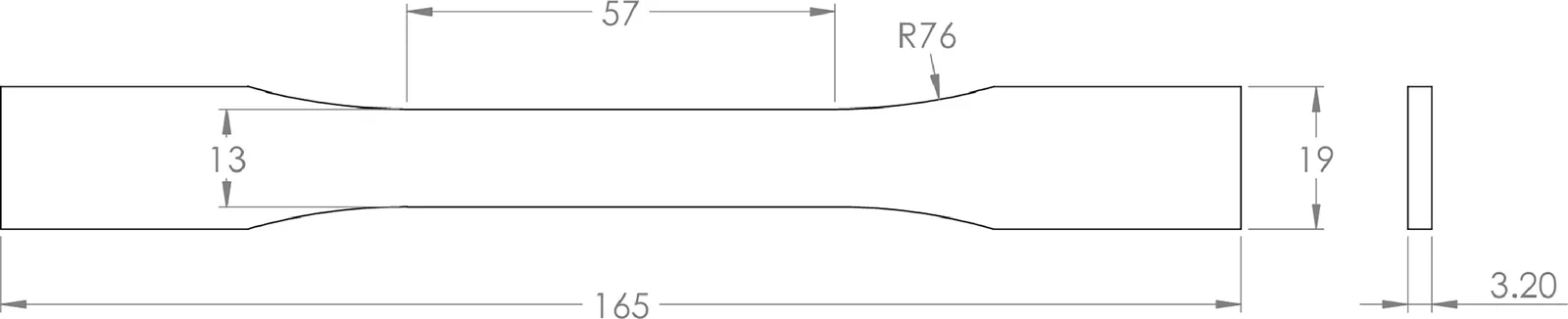
ASTM D638 Type I tensile specimen (dimensions in mm).

As the mechanical properties of FDM parts are strongly governed by the chosen printing parameters [12], the PLA+ material was characterized by uniaxial tensile testing following the ASTM D638 standard [13]. Six Type I tensile specimens, three oriented horizontally and three oriented vertically, were printed at 100% infill density [3]. Fig. 4 shows the dimensions of these specimens. The testing was conducted using a Salt ST-1002 universal testing machine with a 50 kN load cell and a crosshead speed of 5 mm/min. The density of each tensile specimen was obtained from its measured mass and volume, and strain was obtained from crosshead displacement.

**Fig. 5:**
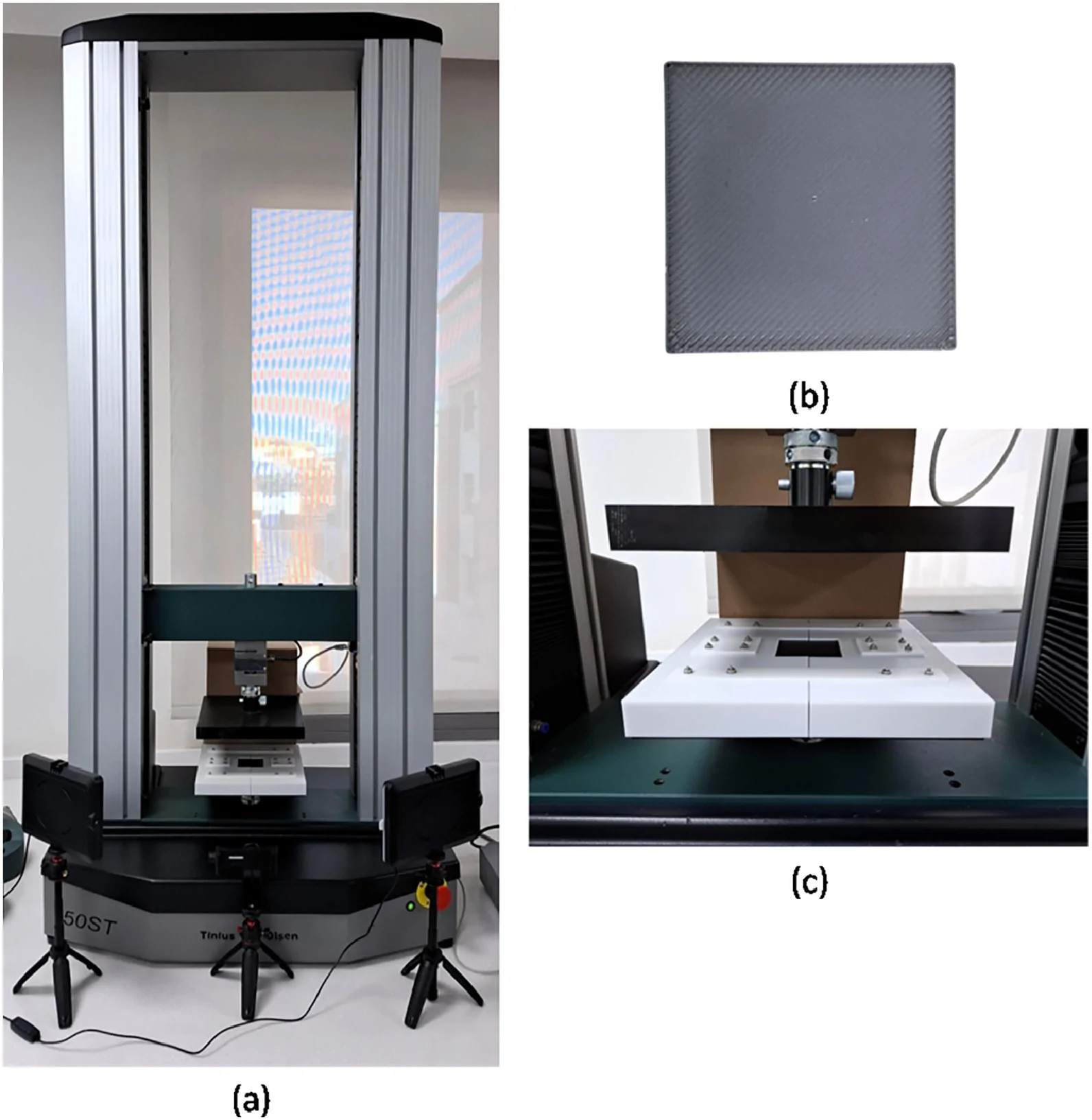
(a) Universal testing machine Tinius Olsen 50ST; (b) base; (c) fixture.

The crashworthiness of the printed specimens was assessed using quasi-static axial compression tests on a Tinius Olsen 50ST universal testing machine equipped with a 50 kN load cell (Fig. 5a). A separate 50 mm × 50 mm × 2 mm base was printed (Fig. 5b), and the net specimen mass was found by subtracting this base mass from the combined mass of the specimen and base. Each specimen was placed between two parallel flat platens, the lower of which carried a printed fixture with a matching 50 mm × 50 mm × 2 mm recess that seated the base and prevented lateral movement during loading (Fig. 5c). Following comparable PLA-based energy absorbers, the upper platen advanced downward at a constant 5 mm/min [14], and the stroke was capped at 50 mm [8]. Force–displacement data were logged throughout the compression test, and sequential images were captured at 5 mm displacement intervals to document the progression of deformation. The integrated base did not deform in any of the tests and therefore did not contribute to the recorded force–displacement response, and no force correction was required. A specimen was included in the dataset only if it was free of visible printing defects and produced a complete, valid test record; specimens not meeting these conditions were excluded, reprinted, and retested under identical conditions. The two baseline configurations (STWC and N-SCMC) were each tested in triplicate to characterize repeatability, whereas the 40 LHS configurations were each tested as a single replicate, consistent with space-filling sampling practice for surrogate and machine-learning modeling [15].

The crashworthiness of each specimen was assessed through several indicators derived from its axial force–displacement response: CE, $E_{T}$, PCF, MCF, SEA, and CFE. PCF is the maximum recorded force, and the remaining indicators are evaluated using Eqs. (2)–(6) [8]:

(2) $$CE=\frac{\delta_{max}}{L}$$

(3) $$E_{T}=\int_{0}^{\delta_{max}}F\;d\delta$$

(4) $$MCF=\frac{E_{T}}{\delta_{max}}$$

(5) $$SEA=\frac{E_{T}}{m}$$

(6) $$CFE=\frac{MCF}{PCF}$$

where δ is the crush displacement, $\delta_{\text{max}}$ is the effective crush displacement, L is the absorber length, F is the instantaneous crushing force, and m is the specimen mass. The effective crush displacement $\delta_{max}$ is taken as the displacement at the onset of densification, identified by scanning each force–displacement curve backward from the end of the test to the point where the slope first turns negative, or as the 50 mm maximum stroke if densification does not occur within the test [8].

## Limitations

All specimens were fabricated from a single PLA+ filament grade using fixed printing parameters on one FDM printer model, and the effects of print parameter variation are not captured. The dataset is limited to quasi-static loading conditions; dynamic or impact loading data are not included, and strain-rate effects on crashworthiness performance are therefore not captured. No compliance correction was applied to the reported data. No extensometer was used in the tensile tests. Per-specimen dimensional tolerances were not measured; the STL files carry the as-designed geometry, and as-printed deviations arise from the FDM process. The 40 LHS-generated configurations were each tested as a single replicate; therefore, per-configuration measurement variance is not available. The geometric design space is bounded by the specified ranges of the four design variables, and extrapolation beyond these ranges is not supported by the data.

## Ethics Statement

The authors affirm that they followed the ethical criteria necessary for publishing in Data in Brief and that this study did not include human volunteers, animal trials, or data gathered from social media platforms.

## Credit Author Statement

**Ahmed Saber:** Resources, Data curation, Writing, Original draft preparation, and Writing-Reviewing; **Murat Altin:** Methodology, Writing-Reviewing and Editing; **Erdem Acar:** Methodology, Writing-Reviewing and Editing, Supervision; **Mehmet Ali Güler:** Resources, Conceptualization, Methodology, Writing-Reviewing and Editing, Visualization.

## Data Availability

Mendeley DataQuasi-static compression test dataset of sinusoidally-corrugated multi-cell PLA+ energy absorbers fabricated by fused deposition modeling (FDM) (Original data).
